# Supplementary material for: Maneuverability of the Scope and Instruments within Three Different Single-Incision Laparoscopic Ports: An Experimental Pilot Study
Source: Animals (Basel). 2021 Apr 26;11(5):1242. doi: 10.3390/ani11051242 (PMC8145893; doi:10.3390/ani11051242)
Supplement: Supplementary file 1 [file animals-11-01242-s001.zip › animals-1141915 - supplementary.pdf]

Table S1 - AREA

mm^2	AREA	AREA (theoretical)
Sil_1ENDO_0Werkzeug_Volumen1.trc	9,8817E+04	1,9349E+04
Sil_1ENDO_0Werkzeug_Volumen2.trc	1,0092E+05	1,8880E+04
Sil_1ENDO_0Werkzeug_Volumen3.trc	8,9092E+04	1,7717E+04
Sil_1ENDO_0Werkzeug_Volumen4.trc	1,0393E+05	2,1626E+04
Sil_1ENDO_0Werkzeug_Volumen5.trc	1,0279E+05	1,9532E+04
Sil_1ENDO_0Werkzeug_Volumen6.trc	1,1082E+05	2,2206E+04
Sil_1ENDO_1Werkzeug_Volumen1.trc	7,6476E+04	1,5976E+04
Sil_1ENDO_1Werkzeug_Volumen2.trc	8,6354E+04	1,6423E+04
Sil_1ENDO_1Werkzeug_Volumen3.trc	9,0168E+04	1,6584E+04
Sil_1ENDO_1Werkzeug_Volumen4.trc	7,3818E+04	1,5757E+04
Sil_1ENDO_1Werkzeug_Volumen5.trc	9,1606E+04	1,8768E+04
Sil_1ENDO_1Werkzeug_Volumen6.trc	1,0198E+05	1,9027E+04
Handschuh_1ENDO_0Werkzeug_Volumen1.trc	1,2938E+05	2,6623E+04
Handschuh_1ENDO_0Werkzeug_Volumen2.trc	1,5056E+05	3,2901E+04
Handschuh_1ENDO_0Werkzeug_Volumen3.trc	1,6547E+05	3,7958E+04
Handschuh_1ENDO_0Werkzeug_Volumen4.trc	1,7133E+05	3,6074E+04
Handschuh_1ENDO_0Werkzeug_Volumen5.trc	1,6331E+05	3,3639E+04
Handschuh_1ENDO_0Werkzeug_Volumen6.trc	1,4759E+05	3,2435E+04
Handschuh_1ENDO_1Werkzeug_Volumen1.trc	1,6321E+05	4,0691E+04
Handschuh_1ENDO_1Werkzeug_Volumen2.trc	2,1855E+05	4,8895E+04
Handschuh_1ENDO_1Werkzeug_Volumen3.trc	2,2527E+05	4,6659E+04
Handschuh_1ENDO_1Werkzeug_Volumen4.trc	2,2895E+05	4,6172E+04
Handschuh_1ENDO_1Werkzeug_Volumen5.trc	2,3042E+05	5,1532E+04
Handschuh_1ENDO_1Werkzeug_Volumen6.trc	2,3028E+05	5,0036E+04
'ENDO_1ENDO_0Werkzeug_Volumen1.trc'	1,7112E+04	4,4374E+03
'ENDO_1ENDO_0Werkzeug_Volumen2.trc'	1,9309E+04	6,0771E+03
'ENDO_1ENDO_0Werkzeug_Volumen3.trc'	1,8315E+04	4,6907E+03
'ENDO_1ENDO_0Werkzeug_Volumen4.trc'	2,2240E+04	6,1683E+03
'ENDO_1ENDO_0Werkzeug_Volumen5.trc'	2,0334E+04	4,9348E+03
'ENDO_1ENDO_0Werkzeug_Volumen6.trc'	2,3321E+04	6,6813E+03
'ENDO_1ENDO_1Werkzeug_links_Volumen1.trc'	1,0875E+04	4,5012E+03
'ENDO_1ENDO_1Werkzeug_links_Volumen2.trc'	9,3906E+03	2,8002E+03
'ENDO_1ENDO_1Werkzeug_links_Volumen3.trc'	8,6045E+03	1,9831E+03
'ENDO_1ENDO_1Werkzeug_links_Volumen4.trc'	9,3207E+03	2,5254E+03
'ENDO_1ENDO_1Werkzeug_links_Volumen5.trc'	8,4367E+03	2,9658E+03
'ENDO_1ENDO_1Werkzeug_links_Volumen6.trc'	8,8453E+03	3,0389E+03
'ENDO_1ENDO_1Werkzeug_mitte_Volumen1.trc'	1,0976E+04	2,8818E+03
'ENDO_1ENDO_1Werkzeug_mitte_Volumen2.trc'	7,6156E+03	2,7920E+03
'ENDO_1ENDO_1Werkzeug_mitte_Volumen3.trc'	8,6923E+03	2,4690E+03
'ENDO_1ENDO_1Werkzeug_mitte_Volumen4.trc'	9,7879E+03	2,3212E+03
'ENDO_1ENDO_1Werkzeug_mitte_Volumen5.trc'	9,1007E+03	3,2894E+03
'ENDO_1ENDO_1Werkzeug_mitte_Volumen6.trc'	6,4189E+03	2,0823E+03
'ENDO_1ENDO_1Werkzeug_rechts_Volumen1.trc'	8,0837E+03	5,9467E+14
'ENDO_1ENDO_1Werkzeug_rechts_Volumen2.trc'	6,0273E+03	4,6289E+03
'ENDO_1ENDO_1Werkzeug_rechts_Volumen3.trc'	6,3307E+03	3,1245E+03
'ENDO_1ENDO_1Werkzeug_rechts_Volumen4.trc'	7,2072E+03	4,4502E+03
'ENDO_1ENDO_1Werkzeug_rechts_Volumen5.trc'	7,9330E+03	4,9381E+03
'ENDO_1ENDO_1Werkzeug_rechts_Volumen6.trc'	6,5670E+03	5,2657E+03

Table S2 - VOLUME

mm^3	Volume	Volume(theoretical)
Sil_1ENDO_0Werkzeug_Volumen1.trc	8,428E+06	1,238E+07
Sil_1ENDO_0Werkzeug_Volumen2.trc	9,263E+06	1,373E+07
Sil_1ENDO_0Werkzeug_Volumen3.trc	8,060E+06	1,242E+07
Sil_1ENDO_0Werkzeug_Volumen4.trc	9,197E+06	3,972E+09
Sil_1ENDO_0Werkzeug_Volumen5.trc	8,284E+06	1,200E+07
Sil_1ENDO_0Werkzeug_Volumen6.trc	9,196E+06	1,297E+07
Sil_1ENDO_1Werkzeug_Volumen1.trc	1,010E+07	1,777E+07
Sil_1ENDO_1Werkzeug_Volumen2.trc	1,161E+07	1,865E+07
Sil_1ENDO_1Werkzeug_Volumen3.trc	1,166E+07	1,785E+07
Sil_1ENDO_1Werkzeug_Volumen4.trc	9,698E+06	1,664E+07
Sil_1ENDO_1Werkzeug_Volumen5.trc	1,261E+07	1,876E+07
Sil_1ENDO_1Werkzeug_Volumen6.trc	1,567E+07	2,165E+07
ENDO_1ENDO_0Werkzeug_Volumen1.trc	1,247E+06	3,316E+06
ENDO_1ENDO_0Werkzeug_Volumen2.trc	1,111E+06	3,917E+06
ENDO_1ENDO_0Werkzeug_Volumen3.trc	1,642E+06	3,996E+09
ENDO_1ENDO_0Werkzeug_Volumen4.trc	1,282E+06	3,512E+06
ENDO_1ENDO_0Werkzeug_Volumen5.trc	1,202E+06	2,325E+06
ENDO_1ENDO_0Werkzeug_Volumen6.trc	1,541E+06	4,530E+06
ENDO_1ENDO_1Werkzeug_links_Volumen1.trc	1,087E+06	3,841E+06
ENDO_1ENDO_1Werkzeug_links_Volumen2.trc	1,027E+06	4,529E+06
ENDO_1ENDO_1Werkzeug_links_Volumen3.trc	9,955E+05	3,521E+06
ENDO_1ENDO_1Werkzeug_links_Volumen4.trc	1,016E+06	3,678E+06
ENDO_1ENDO_1Werkzeug_links_Volumen5.trc	9,308E+05	3,668E+06
ENDO_1ENDO_1Werkzeug_links_Volumen6.trc	9,520E+05	2,771E+06

Table S3 - ANGLES

w/o Instrument	X-links	X-rechts	Y-links	Y-rechts
Endo1	15,0	16,0	21,0	23,0
Endo3	22,0	22,0	21,0	22,0
Endo4	21,0	27,0	25,0	25,0
Endo5	22,0	25,0	24,0	24,0
Endo6	23,0	26,0	25,0	25,0
SILS1	57,0	55,0	61,0	61,0
SILS2	53,0	54,0	56,0	57,0
SILS3	52,0	53,0	52,0	52,0
SILS4	59,0	61,0	56,0	56,0
SILS5	61,0	61,0	56,0	57,0
SILS6	63,0	61,0	59,0	59,0
Handschuh1	63,0	64,0	53,0	51,0
Handschuh2	68,0	66,0	63,0	63,0
Handschuh3	63,0	64,0	66,0	70,0
Handschuh4	63,0	66,0	69,0	74,0
Handschuh5	68,0	66,0	69,0	72,0
Handschuh6	67,0	70,0	66,0	72,0
